# Understanding the Molecular Dynamics of Dual Crosslinked Networks by Dielectric Spectroscopy

**DOI:** 10.3390/polym13193234

**Published:** 2021-09-24

**Authors:** Saul Utrera-Barrios, Reyes Verdugo Manzanares, Javier Araujo-Morera, Sergio González, Raquel Verdejo, Miguel Ángel López-Manchado, Marianella Hernández Santana

**Affiliations:** Institute of Polymer Science and Technology (ICTP-CSIC), Juan de la Cierva 3, 28006 Madrid, Spain; sutrera@ictp.csic.es (S.U.-B.); reyes@ictp.csic.es (R.V.M.); jaraujo@ictp.csic.es (J.A.-M.); sergio@ictp.csic.es (S.G.); r.verdejo@csic.es (R.V.); lmanchado@ictp.csic.es (M.Á.L.-M.)

**Keywords:** nitrile rubber, metal oxides, ionic crosslinks, dual networks, molecular dynamics, dielectric spectroscopy

## Abstract

The combination of vulcanizing agents is an adequate strategy to develop multiple networks that consolidate the best of different systems. In this research, sulfur (S), and zinc oxide ( ZnO) were combined as vulcanizing agents in a matrix of carboxylated nitrile rubber (XNBR). The resulting dual network improved the abrasion resistance of up to ~15% compared to a pure ionically crosslinked network, and up to ~115% compared to a pure sulfur-based covalent network. Additionally, the already good chemical resistance of XNBR in non-polar fluids, such as toluene and gasoline, was further improved with a reduction of up to ~26% of the solvent uptake. A comprehensive study of the molecular dynamics was performed by means of broadband dielectric spectroscopy (BDS) to complete the existing knowledge on dual networks in XNBR. Such analysis showed that the synergistic behavior that prevails over purely ionic vulcanization networks is related to the restricted motions of rubber chain segments, as well as of the trapped chains within the ionic clusters that converts the vulcanizate into a stiffer and less solvent-penetrable material, improving abrasion resistance and chemical resistance, respectively. This combined network strategy will enable the production of elastomeric materials with improved performance and properties on demand.

## 1. Introduction

Elastomers usually undergo a crosslinking process of their polymeric chains (known as vulcanization), which gives them their characteristic elastic behavior. Typically, the vulcanizing agent depends on the elastomer nature, but sulfur is the most widespread used in diene rubbers, forming covalent crosslinks. Sulfur vulcanization allows a precise control over material processing. By varying the proportions of sulfur and of the rest of the ingredients in the vulcanization system (accelerants and activators), networks can be obtained on demand. Peroxide vulcanization is also widely used in rubber matrices for the creation of covalent networks [1].

Special synthetic elastomers such as carboxylated rubbers admit other vulcanization agents like metal oxides (mainly zinc oxide, ZnO, and magnesium oxide, MgO) that generate ionic crosslinks (ion pair) [2]. The rise of carboxylated elastomers as promising elastomeric materials was a consequence of the early work of Brown and Gibbs [3]. In 1955, they presented the first study of properly crosslinked carboxylated elastomers, considering the role of the carboxylic group in the vulcanization. At that time, the carboxylation of elastomers was a novel synthesis proposal that opened the possibility of an alternative crosslinking to that of sulfur. The benefits of the carboxylic group over different properties that were being discovered were adequately collected in multiple reviews of the literature [2,4]. However, it was not until the work of Eisenberg, in which a molecular model (currently in vigor) was presented with the aim of understanding ionic crosslinking as a complex and fascinating structure. According to the Eisenberg model [5,6], the ion pairs are capable of regrouping in higher-order structures known as multiplets and the latter in clusters, which act as supramolecular temporary crosslinking points [7,8]. Despite being more complex to control on an industrial scale, these structures tend to give better mechanical performance than covalent based networks [9,10,11]. With the appearance of carboxylated nitrile rubber (XNBR), and its better performance in multiple properties compared to its original rubber (nitrile rubber, NBR), numerous studies have focused on enhancing its characteristics and improving its processing. Bhowmick et al. have pioneered studies on the structure–properties relationship of XNBR [12], as well as comparative research with NBR from the point of view of vulcanization [13]. Since then, the use of different systems in XNBR has been a constant since each type of crosslink (ionic and covalent) has a particular contribution on the properties of the rubber, and together, they deliver the best of both networks.

Ibarra et al. carried out the vulcanization of XNBR with various ionic network promoters, such as MgO [14], calcium oxide (CaO) [15], and zinc peroxide ( ZnO_2_) [16,17], which was also combined with a secondary covalent network [9,18]. They observed that these systems could produce good mechanical properties and certain stiffness but require high proportions of material, particularly ZnO_2_. Later, Mora-Barrantes et al. [11] reported the combination of dual networks in an XNBR matrix with MgO (ionic) and dicumyl peroxide (DCP, covalent); however, in this case, the covalent network worsened the mechanical performance (modulus at low deformations, tensile strength and tear resistance) obtained with the purely ionic compound at room temperature (RT) but the presence of the covalent network (-C-C-) gave greater stability to the ionic domains at high temperatures. Recently, Krzemińska et al. [19] also prepared individual and dual networks in XNBR compounds based on MgO (ionic) and sulfur (covalent) with unmodified graphene oxide (GO) and carboxylated-GO (GO-COOH). They obtained substantial improvements in some barrier properties due to the existence of the combined network and the successful functionalization of the filler.

Most of the studies in the literature have explained the behavior of their networks through dynamic mechanical analysis (DMA). Few attempts have also been made to explain the nature of covalent and ionic networks by means of broadband dielectric spectroscopy (BDS), which is a powerful, precise, and complementary technique that would allow a better understanding of the molecular dynamics of these complex systems in broad ranges of frequency and temperature [20,21,22,23,24,25]. Thus, a comprehensive study of the dual networks and their respective comparison with the individual networks in XNBR by BDS is still pending.

The objective of this research was to study the molecular dynamics of dual networks to understand their effect on the rubber properties. For this, a three-stage process was followed. In the first, three purely ionic compounds were prepared by varying the ZnO content. In the second stage, a covalent compound (only vulcanized with sulfur) was developed with the same crosslink density as one of the ionic compounds. This strategy enabled a direct comparison of the behavior of both networks. In the third stage, dual networks combining ionic and covalent crosslinks were developed. In this stage, ZnO acted as an ionic vulcanization agent, as well as an activator of the sulfur vulcanization (creating a dual network). In all cases, the rheometric properties were studied and correlated with the tensile properties, and with two of the main characteristics for most XNBR applications: chemical and abrasion resistance. Finally, an exhaustive study by means of BDS was carried out with the intention of correlating the molecular dynamics of the dual networks with their physical performance and thus complement the existing knowledge on carboxylated elastomers.

## 2. Materials and Methods

### 2.1. Materials and Compounding

Carboxylated nitrile rubber (XNBR, KRYNAC X 750) with 27 wt. % of acrylonitrile and 7 wt. % of carboxylic groups was kindly supplied by Arlanxeo (Maastricht, The Netherlands) and used as a rubber matrix. Zinc oxide ( ZnO), sulfur (S), and zinc stearate (ZnSt) were acquired from Merck (Darmstadt, Germany), stearic acid (SA) from Alfa Aesar (Kandel, Germany) and *n*-cyclohexyl-2-benzothiazole sulfenamide (CBS) from Biosynth Carbosynth (Berkshire, UK). Toluene (ACS reagent, ≥99.5%) from Merck (Darmstadt, Germany), gasoline 95 and motor oil from Repsol (Madrid, Spain) were used to evaluate the chemical resistance. All products were used as received. The prepared formulations are summarized in Table 1.

All ingredients were mixed in a two-roll mill (MGN-300S, from Comerio Ercole S.P.A., Busto Arsizio, Italy) during 25 min. The mixing protocol is summarized in Table 2. Each compound was vulcanized by compression molding in a hydraulic press (TP300, from Gumix, Barcelona, Spain) according to its curing time at 90% of the maximum torque (t_90_) obtained from the curing curves. At least 24 h after molding, the vulcanized samples were cut with the geometries required for each characterization technique.

### 2.2. Characterization

#### 2.2.1. Rheometric Properties

The rheometric properties of the compounds were obtained through the study of the curing curves. An oscillating disk rheometer (Rubber Process Analyzer, RPA 2000, from Alpha Technologies, Akron, OH, USA) was used during a fixed time of 120 min, with an oscillation arc of 0.5°, a frequency of 1.6 Hz and a temperature of 160 °C. The values of maximum torque (*M*_H_), minimum torque (*M*_L_), scorch time (*t*s_2_), and *t*_90_ from the elastic component ($S^{\prime }$) were recorded. Curing rate (CR) was obtained from the slope of the ascending linear zone of the curing curves.

#### 2.2.2. Crosslink Density

The crosslink density $\left(\nu \right)$ expressed in mol cm^−3^ was determined by the swelling method. Five squared section samples of each formulation of approximately 1 cm^2^ (area) and 2 mm (thickness) were immersed in toluene for 72 h at room temperature, extracted and dried until the solvent evaporated. The mass of each sample was determined before immersion ($m_{1}$), swollen after 72 h ($m_{2}$), and after evaporation of the absorbed solvent ($m_{3}$). The crosslink density was calculated according to Equation (1):

(1) $$\nu =\frac{1}{2}\left(\frac{\rho_{r}}{M_{c}}\right)$$

The Flory–Rehner expression [26,27] was used to determine the relationship between the density of the compound ($\rho_{r}$) and the molecular weight between crosslinks ($M_{c}$), according to Equation (2):

(2) $$\frac{\rho_{r}}{M_{c}}=-\frac{\ln\left(1-V_{r}\right)+V_{r}+\chi V_{r}{}^{2}}{V_{S}\left(V_{r}{}^{\frac{1}{3}}-\frac{V_{r}}{2}\right)}$$

where χ is the Flory–Huggins interaction parameter between the XNBR and the solvent (estimated as 0.4132 + 0.4341$V_{r}$) [23], $V_{o}$ is the molar volume of toluene (106.20 cm^3^ mol^−1^), and $V_{r}$ is the volume fraction of rubber in the compound, calculated following Equation (3):

(3) $$V_{r}=\frac{\frac{m_{3}}{\rho_{r}}-V_{f}}{\frac{m_{3}}{\rho_{r}}-V_{r}+\frac{m_{2}-m_{3}}{\rho_{s}}}$$

where $V_{f}$ is the volume fraction of fillers, if any, and $\rho_{s}$ is the toluene density (0.867 g cm^−3^). For this and all calculations, the mean values and their respective errors are reported.

#### 2.2.3. Attenuated Total Reflectance-Infrared Spectroscopy (ATR-IR)

Infrared spectra were obtained directly on raw and vulcanized samples, and in ZnO and ZnSt powder using the ATR-IR mode. A wavenumber scan was made from 450 cm^−1^ to 4000 cm^−1^ with a resolution of 4 cm^−1^ and 4 scans per spectrum in an ATR-IR spectrometer (Spectrum Two, from PerkinElmer, Waltham, MS, USA).

#### 2.2.4. Tensile Properties

The tensile test was carried out on five dog-bone test samples (type III) for each compound. A universal testing machine (3366, from Instron, Grand Rapids, Michigan, United States of America) was used at room temperature, with a crosshead initial distance of 35 mm and a crosshead speed of 200 mm min^−1^, following the ASTM D412 (2013) standard [28]. The values of the stress at 100% and 300% deformation (*M*_100_ and *M*_300_, respectively), the tensile strength (maximum stress at the break point, $\sigma_{b}$), and the elongation at break ($\varepsilon_{b}$) were recorded.

#### 2.2.5. Abrasion Resistance

Abrasion resistance index (ARI_A_) was calculated following an ASTM D5963 (2015) standard [29]. Method A (non-rotating) was used. Loss in mass after abrasion was determined and converted to volume loss using the density of each rubber sample. The ratio between the volume loss of the sample and that of Standard Rubber # 1 (density of 1.7034 g cm^−3^) tested under the same conditions was expressed in percent. The abrasion test was performed in Abrasimeter DIN. Three samples (13 mm diameter and 15 mm height) of each compound were prepared directly by compression molding.

#### 2.2.6. Chemical Resistance

Five squared section samples of each formulation of approximately 1 cm^2^ (area) and 2 mm (thickness) were immersed in three non-polar solvents (toluene, gasoline, and motor oil) during 72 h at room temperature, and then were extracted and weighted. Chemical resistance was followed as an indirect measure of the mass change (Δm), according to Equation (4):

(4) $$\Delta m\left(\%\right)=\left(\frac{m_{f}-m_{i}}{m_{i}\;}\right)100$$

where $m_{i}$ is the mass before immersion in the solvents, and $m_{f}$ is the mass of the swollen specimen after 72 h of immersion.

#### 2.2.7. Dynamic Mechanical Analysis (DMA)

A temperature sweep from −100 °C to 150 °C, with a heating rate of 2 °C min^−1^, was carried out in a DMA analyzer (DMA Q800 from TA Instruments, New Castle, DW, USA). An amplitude of 15 μm and a frequency of 1 Hz were set in a tension mode. With this technique, special attention was paid to the loss factor (or tan(δ)), defined as the ratio between the imaginary ($E^{\prime \prime }$ or loss module) and real ($E^{\prime }$ or storage module) component of the complex module ($E^{*}$), calculated according to Equation (5):

(5) $$\tan(\delta )=\frac{E^{\prime \prime }}{E^{\prime }}$$

This loss factor allows for easily observing the transitions of this material, presenting a maximum value in each one of them. In the case of glass transition temperature, *T*_g_, it is possible to find it up to 17 °C above the thermal *T*_g_ detected by differential scanning calorimetry [30].

#### 2.2.8. Differential Scanning Calorimetry (DSC)

The thermal glass transition temperature (*T*_g_) was monitored by DSC in a dynamic mode. The spectra were performed in a calorimeter (DSC-214, from Netzsch, Selb, Germany) under nitrogen flux of 2 mL min^−1^ from −50 °C to 200 °C at 10 K min^−1^ heating rate.

#### 2.2.9. Broadband Dielectric Spectroscopy (BDS)

BDS measurements were carried out on a high-resolution dielectric analyzer (ALPHA from Novocontrol Technologies GmbH, Montabaur, Germany). Films were prepared directly by compression molding and placed between two parallel gold electrodes with a diameter of 30 mm. Frequency (f) sweeps from 10^−1^ Hz to 10^6^ Hz were performed. The range of temperatures studied was from −50 °C to 100 °C with a step of 5 °C. Recording the complex impedance allows the calculation of the complex permittivity $\left(\varepsilon^{*}\right)$, expressed as a function of two components: real $\left(\varepsilon^{\prime }\right)$ and imaginary $\left(\varepsilon^{\prime \prime }\right)$, as seen in Equation (6):

(6) $$\varepsilon^{*}=\varepsilon^{\prime }-i\varepsilon^{\prime \prime }$$

To extract more information, the modulus $\left(M^{*}\right)$ formalism is usually used, also expressed as a function of two components: real $\left(M^{\prime }\right)$ and imaginary $\left(M^{\prime \prime }\right)$, and calculated as the reciprocal of complex permittivity ($1/\varepsilon^{*}$), according to Equation (7):

(7) $$M^{*}=M^{\prime }+iM^{\prime \prime }=\frac{\varepsilon^{\prime }}{{\left(\varepsilon^{\prime }\right)}^{2}+{\left(\varepsilon^{\prime \prime }\right)}^{2}}+i\frac{\varepsilon^{\prime \prime }}{{\left(\varepsilon^{\prime }\right)}^{2}+{\left(\varepsilon^{\prime \prime }\right)}^{2}}$$

Some dielectric relaxations can be described by a Havriliak–Negami (HN) function fit, according to Equation (8):

(8) $$\varepsilon^{*}=\varepsilon_{\infty }+\frac{\varepsilon_{0}-\varepsilon_{\infty }}{{\left(1+{\left(i\omega \tau_{HN}\right)}^{\alpha }\right)}^{\beta }}$$

where $\varepsilon_{0}$ and $\varepsilon_{\infty }$ are relaxed (ω=0) and unrelaxed (ω=∞) dielectric constant values, $\Delta \varepsilon =\varepsilon_{0}-\varepsilon_{\infty }$ is the dielectric strength, α and β are symmetrical and asymmetrical shape parameters, respectively (0<α, β≤1), ω is the angular frequency (ω=2πf), and $\tau_{HN}$ is the mean value of the relaxation time.

A secondary power law function (Equation (9)) is also required to fit the effects of conductivity (σ) on dielectric behavior:

(9) $$\varepsilon^{\prime \prime }={\left(\frac{\sigma_{o}}{\varepsilon_{v}2\pi f}\right)}^{s}$$

where $\sigma_{o}$ is related with the direct current electrical conductivity, $\varepsilon_{v}$ is the dielectric constant of vacuum, and the exponent 0<s<1 depends on the conduction mechanism.

The temperature dependence of some relaxations can be adjusted using the Vogel–Fulcher–Tammann–Hesse (VFT) function, according to Equation (10):

(10) $$\tau_{max}=\tau_{0}\exp\left(\frac{B}{T-T_{0}}\right)$$

where B and $\tau_{0}$ are empirical parameters and $T_{0}$ is the Vogel temperature (generally 30 K–70 K less than *T*_g_ in α relaxation [22]) and $\tau_{max}$ is calculated following Equation (11):

(11) $$\tau_{max}=\frac{1}{2\pi f_{max}}=\tau_{HN}{\left[\sin\left(\frac{\alpha \pi }{2+2\beta }\right)\right]}^{-\frac{1}{\alpha }}{\left[\sin\left(\frac{\alpha \beta \pi }{2+2\beta }\right)\right]}^{\frac{1}{\alpha }}$$

where $f_{max}$ is the frequency at which the maximum of relaxation appears in $\varepsilon^{\prime \prime }\left(f\right)$.

Other relaxations, with different dynamics (especially at low temperatures), can be adjusted following an Arrhenius function, according to Equation (12):

(12) $$\tau_{max}=\tau_{0}\exp\left(\frac{-E_{a}}{RT}\right)$$

that allows the calculation of an activation energy ($E_{a}$). R is the universal gas constant (8.314 J K mol^−1^). All temperatures in Equations (10) and (12) are in absolute scale (K).

## 3. Results

### 3.1. Study of the Individual Crosslinked Networks

In the first stage, we analyzed individual crosslink networks in an XNBR matrix. Three compounds were made with increasing amounts of ZnO (2.5, 5 and 10 phr) and one pure covalent with 1 phr of S. Figure S1 shows the values of crosslink density. The results of 1S and 10 ZnO stand out because of their similarity; thus, they can be considered as equivalent crosslinked networks.

**Figure 1:**
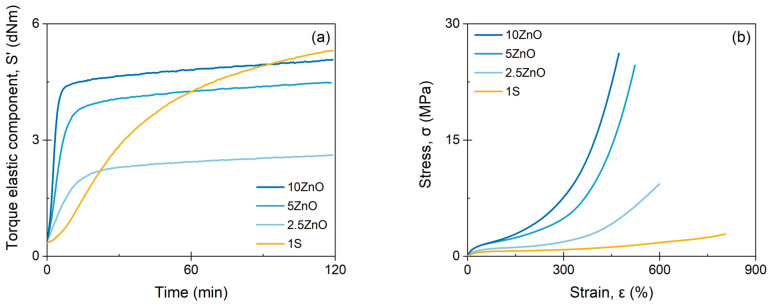
(**a**) Curing curves and (**b**) stress–strain curves of ionic and covalent compounds.

Curing curves (Figure 1a) show that each network has a different behavior during vulcanization; the covalent network draws a less pronounced curve showing a slow vulcanization rate. It is important to mention that the 1S recipe does not contain activators, so very long times are expected to accomplish full vulcanization. In contrast, the ionic network presents a much faster vulcanization rate. In addition, instead of reaching a *plateau*, the ionic network curves have a slightly upward trend (marching modulus), this being a characteristic behavior of these types of crosslinks [8,11]. Table S1 summarizes the data from the curing curves. As expected, the *M*_H_ value increases with the amount of ZnO due to the increase in the ionic crosslinks points in the matrix that cause a greater resistance to shear deformation [14].

In the ionic networks, the crosslinking of the XNBR matrix is carried out according to Equation (13):

(13) $$2\;\mathrm{COOH}+\mathrm{ZnO}\to 2\left({\mathrm{COO}}^{-}\right){\;\mathrm{Zn}}^{2+}+H_{2}O$$

The incorporation of metal ions yields elemental bonds in the matrix, defined as ionic pairs, and originated by the association of the anionic groups COO^−^ and the cations Zn^2+^. The selected XNBR has 7 wt. % of carboxyl groups (-COOH); hence, according to Equation (13), the necessary amount of ZnO to obtain the saturation of these groups in 100 g of XNBR is approximately 6 g. According to the Eisenberg model [6], the ionic pairs in the matrix are capable of forming associations called multiplets. If the proportion of multiplets is high, the ionic domains create clusters that reduce the mobility of the polymeric chains, adding rigidity to the matrix and increasing the tensile strength at room temperature.

Regarding their stress–strain curves, both networks show a very characteristic and different behavior. Despite their equivalent crosslink densities, it can be seen how the ionic network reaches very high values of tensile strength 28 ± 5 MPa with elongation at break of 472 ± 33%, while the covalent network shows a low tensile strength 4.4 ± 0.7 MPa, but high elongation at break 829 ± 54% (Figure 1b). Such a behavior can be related to the bond energy. In covalent bonds, such as monosulfide (-S-), disulfide (-S-S-), polysulfide (-S_x_-), carbon–carbon (-C-C-), among others, the weaker bond tends to give higher tensile strength due to a mechanism of stress dissipation; however, in ionic bonds, the strength of the electrostatic interactions and the formation of clusters that trap polymer chains (stiffening it) have a greater effect on the performance of the material, generating a considerable increase in mechanical resistance with a detriment of the elongation at break [11,31].

Tensile strength and crosslink density increase with ZnO content, showing a significant rise between 2.5 and 5 phr (Table S2). As previously stated, the saturation of the carboxylic groups in the XNBR matrix is reached with 6 phr ZnO. Hence, the ionic clusters in 5 ZnO (close to the saturation point) would explain the increase in mechanical resistance and in crosslink density with respect to the 2.5 ZnO compounds. Meanwhile, the excess of ZnO in the 10 ZnO compound remains in the matrix without forming crosslinks, and it has no relevant effect on tensile properties. Xu et al. [32] reported similar results when the amount of ZnO in the matrices exceeded the saturation point of the COOH groups.

**Figure 2:**
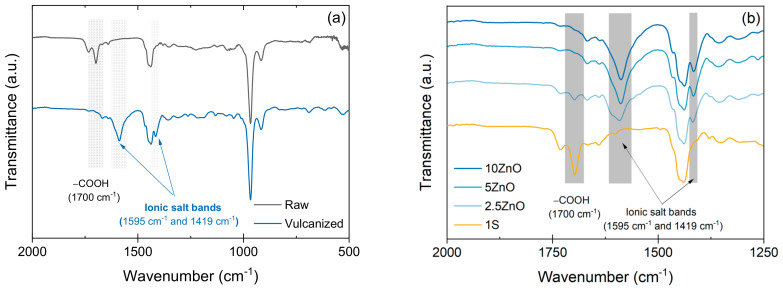
(**a**) ATR-IR spectra of raw and vulcanized 5 ZnO compounds; (**b**) ATR-IR spectra of covalent and ionic compounds.

The structure of each matrix has been verified by infrared spectroscopy (Figure 2). -COOH groups are present in the XNBR matrix, as confirmed by the appearance of a band at 1700 cm^−1^ in the spectrum of a raw sample (associated with carbonyl C=O vibration). When it is vulcanized, the Zn^2+^ ions bind to the COO^−^ groups, forming ionic bonds that are evidenced by the absence of the band at 1700 cm^−1^ and the appearance of two bands: one at 1595 cm^−1^ and another at 1419 cm^−1^, related to the *tetra-* and *hexa-* coordinated zinc carboxylated structure [17,21,32,33]. Figure 2a represents the infrared spectra of the 5 ZnO formulation where the aforementioned signals have been identified.

Figure 2b shows the infrared spectra of the other compounds in the vulcanized state. In the covalent formulation (1S), the -COOH signal appears unchanged at 1700 cm^−1^, indicating that these groups are free in the matrix. In the case of the ionic networks, they all show the characteristic bands at 1595 cm^−1^ and at 1419 cm^−1^. However, for the 2.5 ZnO compound, a small signal also appears at 1700 cm^−1^, associated with free –COOH groups that have not reacted since the amount of ZnO is below its saturation level.

### 3.2. Dual Ionic/Covalent Crosslinked Networks

**Figure 3:**
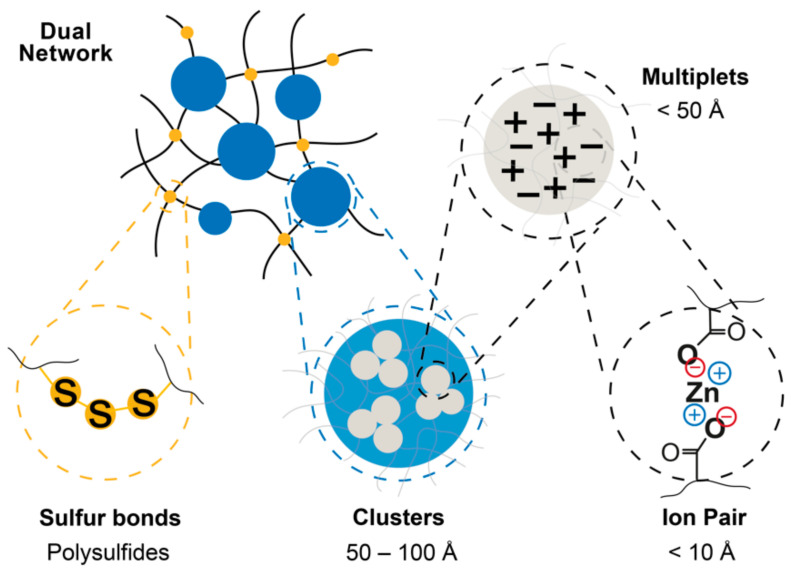
Scheme of the dual crosslinked networks. The scales in this figure are based on estimations in the Eisenberg model for ionomers [6], they may vary between different systems depending on the type of ionic pair and the processing conditions.

Covalent networks crosslinked with sulfur are characterized by their irreversibility. On the opposite side, ionic networks are reversible and have good mechanical properties, but are unstable at temperatures above the ionic transition temperature (*T*_i_); in common rubbers, *T*_g_ < RT < *T*_i_. In accordance with these conditions, efforts have been made to combine the best of both practices, following different strategies [11]. In this section, the effect of combining both networks on the XNBR is studied. An illustrative scheme of the dual networks is shown in Figure 3.

**Figure 4:**
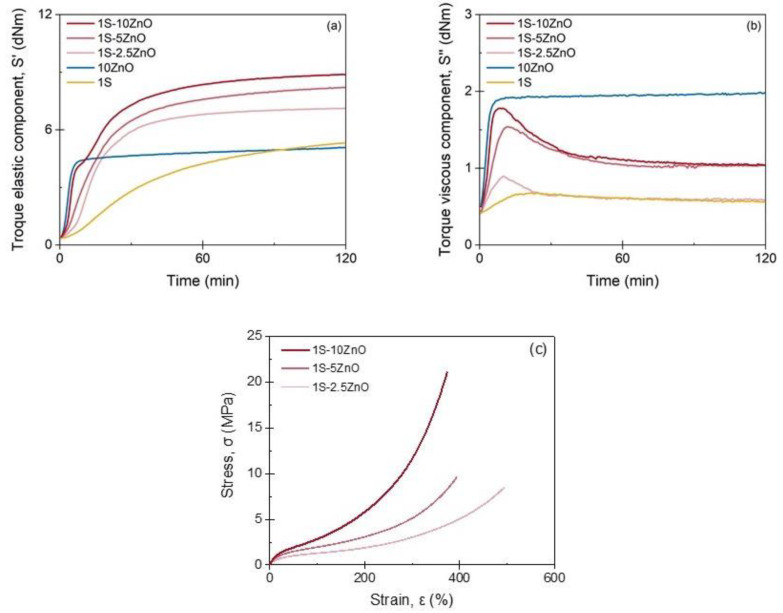
Torque (**a**) elastic and (**b**) viscous components of dual networks compounds (curing curves); (**c**) stress–strain curves of dual networks’ compounds.

The curing curves of the dual crosslinked networks are shown in Figure 4. The individual ionic and sulfur-cured compounds are also included for comparison purposes. It is possible to identify a trend in the dual networks in which M_H_ increases with the amount of ZnO. Two effects seem to be responsible for this behavior. First, a higher content of metal oxide increases the likelihood that metal ions and carboxyl groups would be in close proximity, favoring the formation of the ionic network. Second, part of the ZnO (in presence of SA) acts as an activator of the sulfur vulcanization, generating a covalent network, with a more efficient use of sulfur.

Certain similarities can be seen between the dual networks and the purely ionic one (10 ZnO). The marching modulus for the elastic component ($S^{\prime }$), as well as a maximum in the viscous component ($S^{\prime \prime }$) curve, are characteristics of dual networks [9,10]. An interesting effect is also noticed on the curing curve of the dual compound with the highest ZnO content (1S-10 ZnO). Two slopes are clearly detected in the early stages of the vulcanization, which could be confirming the formation of the two networks. This effect is easily observed in $S^{\prime \prime }$ as the presence of a peak. After the typical increase in $S^{\prime \prime }$, because of the initiation of vulcanization, these nets do not reach a *plateau* but start to descend after reaching a maximum value. The descending zone is associated with the covalent network that increases the elastic component, with a corresponding decrease in the viscous component. This behavior is not detected in individual formulations, such as 1S and 10 ZnO, where $S^{\prime \prime }$ reaches a *plateau* [9,17]. Table S3 shows a summary of the values obtained from the $S^{\prime }$ curves.

The same trend shown in the rheometric curves can be observed in the study of the stress–strain curves and crosslink density (Figure 4c and Table S4). As the amount of ZnO increases, the crosslink density and tensile strength increase, which could be ascribed to a higher number of ionic and sulfur bonds that would eventually act as reinforcing points, increasing the tensile strength.

**Figure 5:**
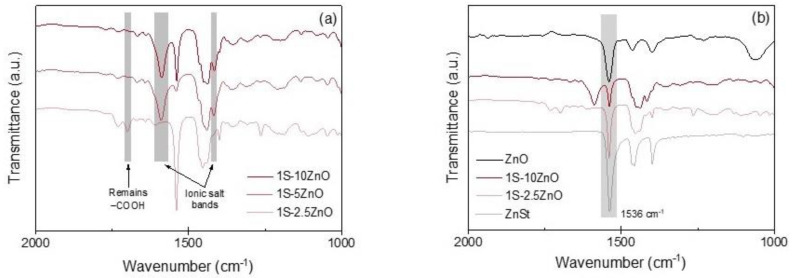
(**a**) ATR-IR spectra of dual networks compounds; (**b**) ATR-IR spectra of 1S-2.5 ZnO and 1S-10 ZnO compounds with ZnO and zinc stearate (ZnSt) powder.

The internal structure of the compounds was studied by ATR-IR. In Figure 5a, the spectra of the dual networks are observed. The bands associated with ionic bonds appear at 1595 cm^−1^ and 1419 cm^−1^ with an increasing amount of ZnO. For the case of 1S-5 ZnO and 1S-10 ZnO, there is enough ZnO to promote ionic vulcanization and form clusters. It is important to mention a band located at 1536 cm^−1^, which is present in all compounds, but stands out to a greater extent for 1S-2.5 ZnO and 1S-10 ZnO. This band coincides with another one in the spectra of pure ZnO and ZnSt powder (Figure 5b). Two plausible explanations can be considered. The prominent band can be related to the excess of ZnO beyond the saturation limit or to the formation of ZnSt, which is a characteristic secondary product of accelerated sulfur-based systems. This could confirm that, even at low metal oxide contents, the formation of the sulfur vulcanization activator complex begins and competes with the formation of ionic crosslinks.

### 3.3. Chemical and Abrasion Resistance of Individual and Dual Networks

**Figure 6:**
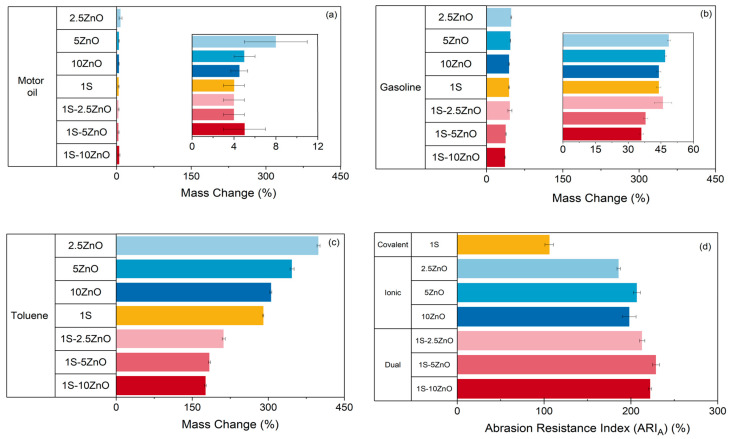
Chemical resistance in (**a**) motor oil, (**b**) gasoline, (**c**) toluene, and (**d**) abrasion resistance index (ARI_A_) of covalent, ionic, and dual compounds (networks).

XNBR has high chemical resistance to non-polar solvents, such as gasoline and motor oil, due to its intrinsic chemical structure influenced by nitrile (-CN) and carboxyl (-COOH) functional groups. These characteristics enable its extensive use in the automotive industry, especially for the manufacture of gaskets and hoses [8]. For the analysis of the chemical resistance, five specimens of each formulation were immersed in three types of solvents: toluene (non-polar aromatic solvent), gasoline 95 (non-polar aliphatic solvent), and motor oil (high viscosity non-polar aliphatic solvent) for 72 h. The results of this test are shown in Figure 6.

Both types of individual crosslinked matrices show similar chemical resistance regardless of the solvent. In general, it is observed that all the formulations have less mass change in the case of motor oil, followed by gasoline and, finally, toluene. Thus, their resistance will be higher for oil and, in contrast, lower for toluene. An increase in chemical resistance is also observed with increasing ZnO content. This behavior may be correlated with the decrease in free volume, which restricts the diffusion of the solvent in the matrix [34,35]. Regarding the dual networks, it is important to highlight that the combination of crosslinks yielded the highest chemical resistance, with reductions of up to ~26% in compound swelling. This can be ascribed to a synergistic effect by the presence of the two types of networks that further hinder the diffusion of the solvent into the matrix. This effect can be observed in other systems with certain similarities such as Interpenetrated Polymeric Networks (IPN) in which two crosslinked nets are mixed, and physically interlaced in a molecular scale [36]. The compound with the best performance was 1S-10 ZnO with a mass change of 176 ± 2% in toluene and 36 ± 1% in gasoline.

Abrasion resistance is the ability of a material to maintain its structure and shape after suffering damage from erosion and/or wear of their surface. This characteristic is important in the case of materials that require a use in which the shape is essential; therefore, the surface resistance would facilitate the shape retention and, thus, its functionality. In XNBR, the functional groups of the matrix originate secondary interactions that form strong bonds, causing high abrasion resistance compared to other rubbers [8]. Even though in chemical resistance the individual crosslinked matrices show a similar behavior, it is in the abrasion resistance where their differences are indisputable. Figure 6d shows that the individual ionic matrix has a higher abrasion resistance index 198 ± 8% than the covalent peer 106 ± 7%. This increase of ~86% can be correlated with previous explanation about the strength of the bonds. In the case of the ionic matrix, the bonds have greater intensity and are stronger than in the case of the sulfur-cured matrix [14,31]. The dual networks also yielded the highest values of abrasion resistance in this research. A maximum is reached for the 1S-5 ZnO compound with an abrasion resistance index of 229 ± 4%. Comparing the values for the ionic and dual formulations, one can observe increments of up to ~15% for the dual matrix and of up to ~115% with respect to the covalent matrix (only with sulfur). This confirms a synergistic effect of both networks on this property.

### 3.4. Understanding the Molecular Dynamics of Dual Networks by Means of BDS

**Figure 7:**
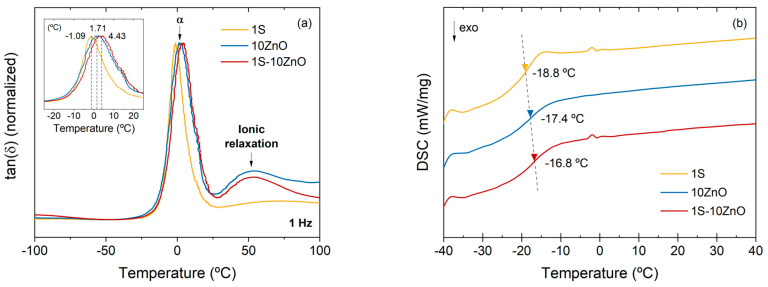
(**a**) DMA and (**b**) DSC spectra for prepared compounds.

A preliminary assessment of the dynamics of the 1S, 10 ZnO, and 1S-10 ZnO compounds was done by DMA. Figure 7a shows two relaxation zones. The *α* relaxation or segmental relaxation is related to the movement of chain segments and associated with the *T*_g_ of rubber (around −1.09 °C for 1S, 1.71 °C for 10 ZnO, and 4.43 °C for 1S-10 ZnO). This relaxation exhibits considerable changes in the shape of the peak, being wider for the ionic (10 ZnO) and dual compound (1S-10 ZnO), versus the covalent one (1S), as well as being shifted to higher temperatures (see Figure 7a inset). This could be associated with a greater restriction of the ionic domains, as a consequence of the chains trapped in the clusters and the performance of these clusters as crosslinking points [37]. A complementary analysis of the glass transition was also performed by DSC (Figure 7b). The values obtained for the thermal *T*_g_ corroborate the trend of the DMA, in which the purely ionic (10 ZnO) and dual (1S-10 ZnO) networks are presented as more restricted systems than the purely covalent (1S) one. The thermal *T*_g_ by DSC was found at −18.8 °C for 1S, −17.4 °C for 10 ZnO, and −16.8 °C for 1S-10 ZnO (all the values reported refer to the midpoints, however, the trend is maintained both at the onset and endpoint).

In addition to *α* relaxation, a second process is detected by DMA (Figure 7a) at higher temperatures: an ionic relaxation around 50 °C that is not present in the covalent formulation (1S), which is an irrefutable proof of the presence of ionic clusters [8,11,21,23,38] in the purely ionic compound, and in the dual one. The differences between 1S-10 ZnO and 10 ZnO could be associated with the compromise between the role of ZnO as vulcanizing agent (for the ionic network) and as a sulfur activator (for the covalent network). In this way, one can corroborate that not all the ZnO in the 1S-10 ZnO would be contributing to ionic bonds, as it does in 10 ZnO.

**Figure 8:**
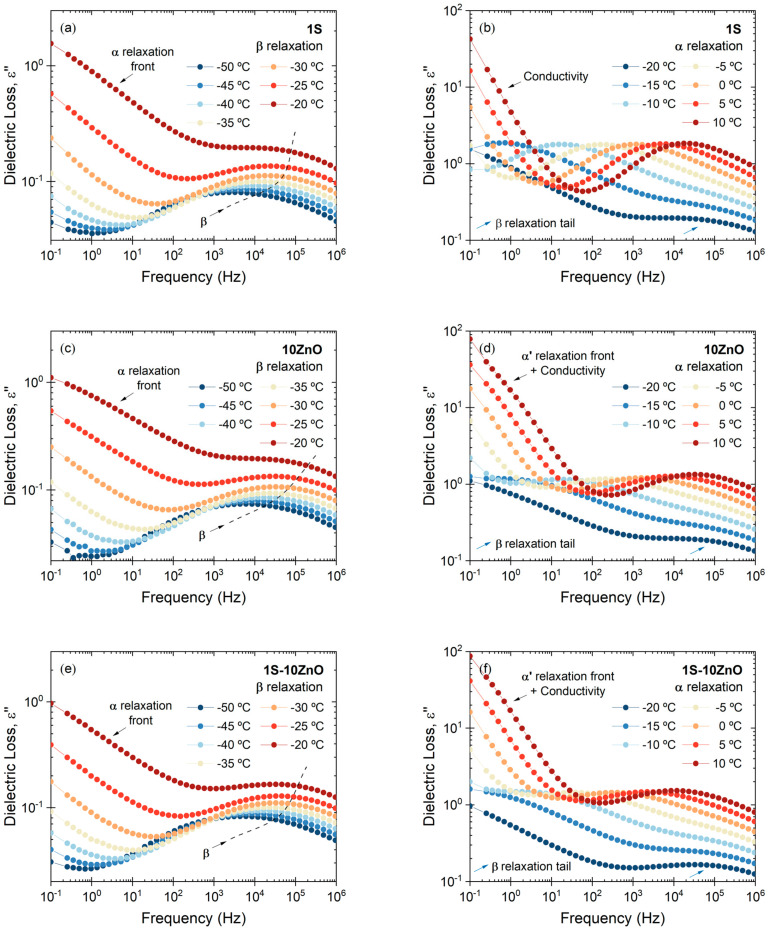
*β* and *α* relaxation in (**a**,**b**) 1S; (**c**,**d**) 10 ZnO; and (**e**,**f**) 1S-10 ZnO.

A systematic analysis was further performed by BDS to gain a deeper knowledge on the dynamics of the three compounds under study. In theory, dielectric and dynamic mechanical analysis should reflect the same motions of the chains and chain segments if the referred motion implies dipole motions; however, some differences in both the intensities and frequencies of the relaxations can be distinguished. In this study, a third weaker relaxation at low temperatures can be detected, *β* relaxation, associated with short-range cooperative movements in the polymeric chain. Figure 8 shows the dielectric loss spectra of the *β* and *α* relaxations at different temperatures and in a wide frequency range. These two relaxations can be confirmed as thermally activated processes, as evidenced by their shift towards higher frequencies with increasing temperature.

**Figure 9:**
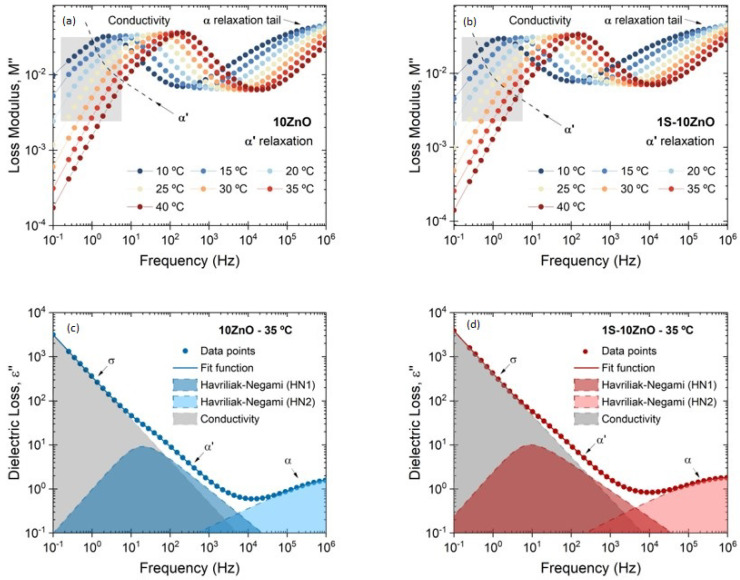
*α*′ relaxation determined by means of modulus ($M^{\prime \prime })$ in (**a**) 10 ZnO and (**b**) 1S-10 ZnO and by means of dielectric loss ($\varepsilon^{\prime \prime }$) in (**c**) 10 ZnO and (**d**) 1S-10 ZnO at 35 °C. The dashed lines in (**c**,**d**) indicate the fittings of Havriliak–Negami and power law function to the experimental data at a selected temperature.

At high temperatures and low frequencies, both the electrode polarization (EP), that partially blocks the charge exchange between the sample and the electrodes, and the accelerated movement of ions generate long capacitances that translate into high dielectric constants and into a considerable increase in conductivity. Such behavior partially masks the third relaxation (ionic relaxation) in the permittivity spectrum ($\varepsilon^{\prime \prime }$). Instead, we used the modulus ($M^{\prime \prime }$) formalism [39] (Figure 9a,b). Both variables describe the same electrical relaxation phenomena; however, under different conditions, a specific form offers more information with respect to the occurring physical processes. For instance, the $M^{\prime \prime }$ formalism “converts” the conductivity into a peak associated with the AC conduction and supresses the effects of DC conduction and EP. The frequency range below the peak in $M^{\prime \prime }$ corresponds to the zone dominated by two effects: EP and the movement of charge carriers over long distances. With the suppression of the contribution of EP, the second effect can be detected from changes in the size and shape of the peak. This physical process, called long-range ionic hopping, is related to large-scale molecular movements between ionic domains: it is the relaxation of the clusters. Previous studies have demonstrated that EP and AC conduction itself are consequences of this process [40]. Other authors [41] have conducted exhaustive and systematically research that report the superiority of the dielectric spectrum plotted in permittivity ($\varepsilon^{*}$) rather than modulus ($M^{*}$) in most of studies for slow process due to motion of molecules/ions; however, in our particular case, the modulus gives us complementary information of the relaxations when the dielectric analysis is conducted, which does not contradict the literature but adds new insights.

The individual ionic compound 10 ZnO exhibits an irregular and asymmetric peak, with an observable shoulder in the low frequency range (1 Hz to 10 Hz) (Figure 9a). This shoulder could prove the presence of a third *α*′ relaxation. Some authors have attributed this new relaxation above the glass transition temperature of the matrix to an individual and secondary relaxation in rubber ionomers identified as *α*′ and associated with the hard ionic domains [21,23,42,43]. In the dual network 1S-10 ZnO compound, this shoulder is less notorious (with respect to 10 ZnO), due to the dual role of ZnO (vulcanizing agent and activator) in the compound (Figure 9b). Once the presence of the third relaxation is detected, it is possible to perform a complex permittivity fitting by using HN functions (for ionic relaxation) and power law (for conductivity), as seen in Figure 9c,d. Other authors have not been able to detect ionic relaxations through dielectric studies, but a third relaxation (at lower temperatures than those observed by DMA, 50 °C–70 °C lower) has been associated with the Maxwell–Wagner–Sillars (MWS) relaxation [44]. This relaxation is characteristic of multiphase systems, where each phase has different dielectric constants and conductivities. If one considers the ionic clusters as a polarizable entity, it will appear as a heterogeneity with respect to the matrix, exhibiting this physical phenomenon. In our case, the data obtained coincide in temperature with those observed by DMA. Hence, either way, both analyses are conclusive on the presence of the ionic phase.

For comparative purposes, and with the intention of discerning the effect of the molecular dynamics on the physical properties of the individual and dual crosslinked compounds, we selected a convenient temperature in which each relaxation was well resolved in the frequency domain.

**Figure 10:**
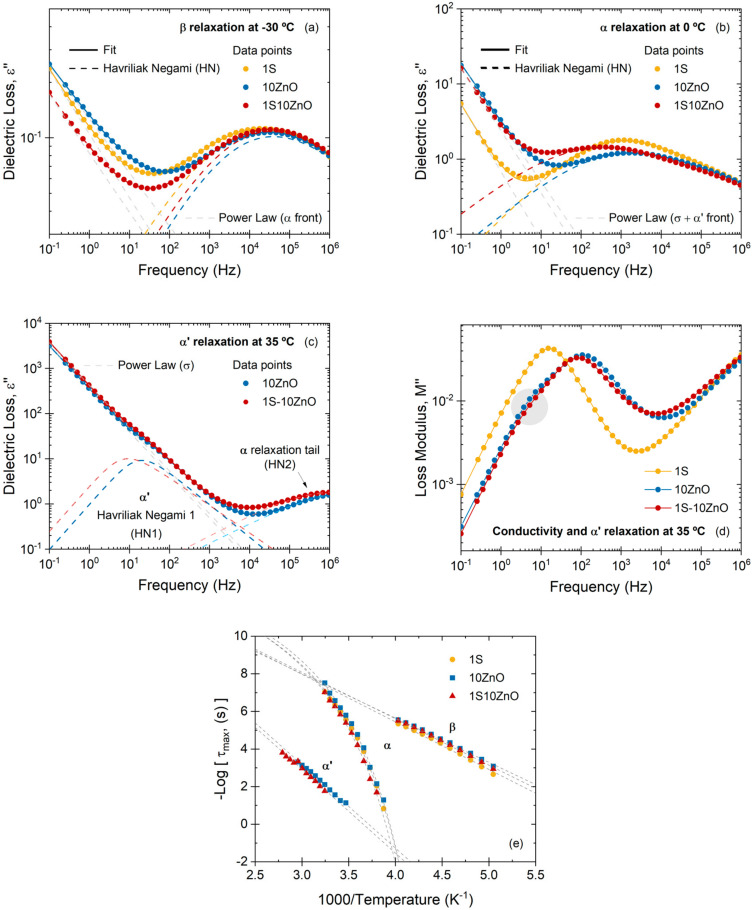
(**a**) *β*, (**b**) *α*, and (**c**) *α*′ relaxation of 1S, 10 ZnO, and 1S-10 ZnO at selected temperatures, (**d**) loss modulus $M^{\prime \prime }$ of compounds at selected temperature, and (**e**) activation diagram of the three relaxations in XNBR compounds. The dashed lines indicate the fittings of (**a**–**c**) Havriliak–Negami and power law function, and (**e**) the Arrhenius and VFT function, to the experimental data.

Figure 10 summarizes all the relaxations at the designated temperature (−30 °C for *β* relaxation, 0 °C for *α* relaxation, and 35 °C for *α*′ relaxation). Starting with the *β* relaxation, it is slightly affected by the type of network (individual or dual) since the covalent (1S) and dual compound (1S-10 ZnO) seem more restricted at this low temperature. This relaxation can be fitted using a HN function and a power law for the high-frequency tail (front) of *α* relaxation. Table S5 summarizes fitting parameters of all relaxations at the chosen temperature. The nature of the crosslinks also does influence both the *α* and *α*′ relaxations, as also seen by DMA. A more restricted network is achieved in the dual system (1S-10 ZnO), evidenced by the shift of the dielectric loss to lower frequencies in the segmental relaxation zone (Figure 10b). This slowing down of the segmental dynamics is a consequence of growing cooperativity. It is evident that, as free volume reduces (due to the crosslinks), more cooperativity is needed to accomplish segmental motions. These molecular dynamics findings are of fundamental interest for understanding, and thus tailoring, properties improvement since the structure of the material can be extracted from the relaxation of polymer chains. Hence, the restrictions on the segmental motions of the rubber chains and the lower free volume convert the vulcanizate into a stiffer and less solvent-penetrable material, improving abrasion resistance and chemical resistance, respectively. *α* relaxation can be fitted using a HN function and a power law for the contributions of conductivity and high-frequency tail (front) of *α*′ relaxation. Moreover, the *α*′ relaxation (Figure 10c) ratifies the formation of ionic domains in the dual network. These ionic domains also provide higher restrictions to the trapped chains within the ionic clusters. At this point, the differences in the conductivity peak in the compounds that have an ionic phase, compared to the purely covalent phase, are also interesting (Figure 10d). This could also be an irrefutable proof of the presence of multiple ions, which increase ionic conductivity, reflected in a peak shift towards higher frequencies [40]. In conclusion, the excellent chemical resistance and abrasion resistance, as well as the retention of tensile strength, confirm the role of these ionic domains in dual networks. To the best of our knowledge, no study has been found in the literature linking the dynamics of these relaxations with those two essential properties in most XNBR applications.

For all temperatures tested, the behavior of the relaxations was evaluated and the frequency at which the maxima appear was recorded. From these values, it was possible to obtain the graph of the logarithm of the relaxation time ($-\log\tau_{max}$) as a function of the inverse of the absolute temperature (1/T) (activation diagram) (Figure 10e). A value of $-\log\tau_{0}\approx 14\;s$ was set to reduce the effect of data fitting over a limited frequency range, according to previous studies [45,46]. With this consideration, a fitting can be made to each relaxation. Table 3 shows all the fitting parameters. The *β* relaxation fits perfectly to the Arrhenius function, which allows the calculation of the relaxation’s activation energy ($E_{a}$). From these values, we can confirm that this relaxation is slightly dependent of the crosslink type. It is observed that the formulations with covalent phase (1S and 1S-10 ZnO) present higher activation energies than the purely ionic phase (10 ZnO).

The *α* relaxation departures from the Arrhenius behavior; it exhibits a curvature at high temperatures adjusting perfectly to the VFT function. This relaxation is shifted to the left, which means a higher temperature requirement for its activation compared to the *β* relaxation. In addition, the ionic networks (10 ZnO and 1S-10 ZnO) are shifted to higher temperatures (increasing values of *T*_0_), showing also different time scales between compounds. This could be explained by the higher restriction in ionic domains, which hinders the motions of rubber chain segments and slows down the *α* relaxation. To deepen the analysis, two quantities can be obtained from the parameters derived from the VFT adjustment. The first of them is known as fragility strength (D), which can be calculated from Equation (14), and is related to the nature of the *T*_g_ (strong or fragile behavior) [47,48]:

(14) $$B=DT_{0}$$

Second, the fragility index (or steepness index, m) [47,48], which is defined as an apparent activation energy and can be estimated according to Equation (15):

(15) $$m={\left.\frac{\partial \log\tau \left(T\right)}{\partial \left(\frac{T_{g}}{T}\right)}\right|}_{T=T_{g}}=\frac{DT_{o}T_{g}}{\ln10{\left(T_{g}-T_{0}\right)}^{2}}\approx 16+\frac{590}{D}$$

In polymeric materials, the strong or fragile behavior is associated with the cooperativity of the segmental chain movements. Thus, highly cooperative materials (fragile, typically with more rigid backbones) will strongly deviate from the Arrhenius behavior and will have low D values (high m), i.e., will require a higher apparent activation energy than those with low cooperativeness [49]. According to the data shown in Table 3, a dependence of the $T_{0}$ values with the type of crosslink is identified. This can also be correlated with the *T*_g_ values seen as the maximum of the $\tan\left(\delta \right)$ curves in DMA (Figure 7). 1S has a lower *T*_g_, followed by 10 ZnO and finally 1S-10 ZnO. Although 1S and 10 ZnO compounds have equivalent crosslink densities with the swelling method, slight differences are detected in *T*_g_ that may be attributed to the nature of crosslink, and not to an effective increase in the number of crosslinking points. According to the above, it is consistent that the values of $T_{0}$ are higher for 10 ZnO and 1S-10 ZnO, and, consequently, the values of D are lower. From the values of m, it can be concluded that, in the dual system, the fragility (low D, high m) is closest to the fragility of 10 ZnO because the dual networks impose similar restrictions on the segmental movements of the chains [45].

The *α*′ relaxation adjusts to the Arrhenius function. An attempt has been made to adjust this relaxation with a VFT function. Although adjustment is possible, with a correlation coefficient ≥0.98, the *T*_0_ values obtained lack physical meaning. Considering the activated character, 1S-10 ZnO deviates from 10 ZnO due to its more restricted network, as reflected in its shifted to higher temperatures. This ionic relaxation could be thought of as a “second glass transition” associated with the hard ionic phase in the rubber matrix that gains mobility with increasing temperature [8]. Some authors have suggested that, in the presence of ZnO, these ionic domains are an individual and differentiated phase of the rubber matrix, which contains a coordinated complex based on the ionic pairs (Zn^2+^ and COO^−^), as well as trapped chains. These domains form a three-dimensional zone. This zone would act as a separate polymer phase from the rest of the matrix, with its own “glass transition temperature” [21] that can be redefined as “ionic transition temperature”. Upon exceeding this temperature, the trapped chains in clusters are released, and the ion pairs acquire mobility due to the ion-hopping mechanism, destroying coordination complexes and ionic clusters. In the dual network, these ionic domains can be surrounded (or even partially constituted) by covalent crosslinks points (S-based) that provide greater rigidity to the system. All these conclusions open a new path to study different effects on combined networks. Varying the size of the ionic domains, the cations involved and/or the proportions of the ingredients of the sulfur crosslinking system will allow for obtaining networks *on demand* for specific applications.

## 4. Conclusions

In this research, we have performed a comprehensive study of ionic (with zinc oxide) and covalent (pure sulfur-based) crosslinked networks, as well as their combination in an XNBR matrix. The results show that by combining both networks it is possible to retain the good tensile strength that characterize ionic crosslinks, with a substantial improvement in two essential properties in most XNBR applications: chemical and abrasion resistance. The synergistic effect between both networks improves the abrasion resistance of up to ~15% compared to a pure ionic network, and up to ~115% compared to a pure sulfur network. It was also possible to further improve the chemical resistance of XNBR in non-polar fluids such as toluene and gasoline. A reduction of up to ~26% of the solvent uptake was achieved. A plausible interpretation to these improvements is given based on the molecular dynamics of the prepared compounds. It was found that the combination of covalent and ionic crosslinks resulted in a more restricted network. Such limitations on the motions of chain segments are responsible for obtaining an XNBR with more stiffness and reduced interaction with the solvent molecules, limiting their diffusion within the rubber matrix, thus improving abrasion and chemical resistance. In conclusion, the combination of different kinds of crosslink networks remains a suitable strategy in carboxylated elastomers such as XNBR, for obtaining properties on demand and for opening new paths in the development of unprecedented rubber products.

## Figures and Tables

**Table 1:** Rubber compounds’ recipes expressed in parts per hundred parts of rubber (phr).

	Ionic Crosslinks			Sulfur Crosslinks	Dual Crosslinks		
Ingredient	2.5 ZnO	5 ZnO	10 ZnO	1S	1S-2.5 ZnO	1S-5 ZnO	1S-10 ZnO
XNBR	100	100	100	100	100	100	100
ZnO	2.5	5	10	0	2.5	5	10
SA	0	0	0	0	1	1	1
CBS	0	0	0	0.25	0.25	0.25	0.25
S	0	0	0	1	1	1	1

**Table 2:** Mixing protocol.

Steps	Time (min)
Mixing Two-Roll Mill, Room Temperature, Friction Ratio of 1:1.15.	
Ionic crosslinks compounds	
Add XNBR	0 min
Add ZnO	5 min
Sulfur crosslinks compounds	
Add XNBR	0 min
Add S	5 min
Add CBS	7 min
Dual crosslinks compounds	
Add XNBR	0 min
Add ZnO	5 min
Add SA	7 min
Add S	9 min
Add CBS	11 min
Make six transversal cuts	15 min
Discharge	25 min

**Table 3:** Arrhenius and VFT fit parameters in activation diagrams.

	1S	10 ZnO	1S-10 ZnO
β relaxation—Arrhenius function			
$E_{a}$ (kJ/mol)	48 ± 2	45 ± 1	47 ± 1
**α relaxation—VFT function**			
B	1669 ±30	1574 ± 29	1649 ± 29
$T_{0}$ (K)	202 ± 1	204 ± 1	205 ± 1
D	8.3 ± 0.2	7.7 ± 0.1	8.0 ± 0.1
m	87 ± 3	93 ± 4	90 ± 3
***α***′ **relaxation—Arrhenius function**			
$E_{a}$ (kJ/mol)	-	85 ± 2	85 ± 3

## Data Availability

The data that support the findings of this study are available on request from the corresponding author, M.H.S.

## Supplementary Materials

The following are available online at https://www.mdpi.com/article/10.3390/polym13193234/s1, Figure S1: Crosslink density of XNBR compounds. Table S1: Rheometric properties of ionic and covalent compounds. Table S2: Tensile properties of covalent and ionic compounds. Table S3: Rheometric properties for dual network compounds. Table S4: Tensile properties of dual network compounds. Table S5: HN fitting parameters of compounds at selected temperatures.
