# Supplementary material for: Understanding the Molecular Dynamics of Dual Crosslinked Networks by Dielectric Spectroscopy
Source: Polymers (Basel). 2021 Sep 24;13(19):3234. doi: 10.3390/polym13193234 (PMC8512226; doi:10.3390/polym13193234)
Supplement: Supplementary file 1 [file polymers-13-03234-s001.zip › polymers-1398130-supplementary.pdf]

SUPPORTING INFORMATION

Understanding the molecular dynamics of dual crosslinked networks by dielectric spectroscopy

Saul Utrera-Barrios[1], Reyes Verdugo Manzanares[1], Javier Araujo-Morera, Sergio González, Raquel Verdejo, Miguel Ángel López-Manchado, Marianella Hernández Santana*

Institute of Polymer Science and Technology (ICTP-CSIC), Juan de la Cierva 3, 28005 Madrid, Spain

[1] S.U.-B. and R.V.M contributed equally to this work

* corresponding author: marherna@ictp.csic.es

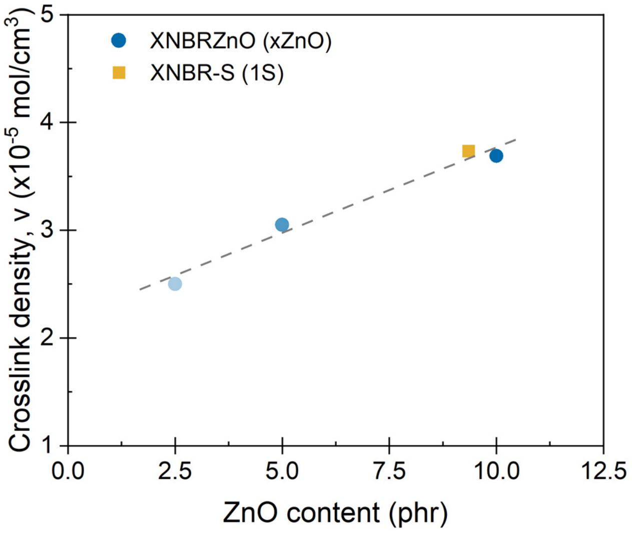

Figure S1. Crosslink density of XNBR compounds: ionically cured varying the amount of ZnO (denoted as 2.5ZnO, 5ZnO and 10ZnO) and sulfur-cured (denoted as 1S).

Table S1. Rheometric properties of ionic and covalent compounds.

	Compounds			
**Parameters**	**2.5ZnO**	**5ZnO**	**10ZnO**	**1S**
M_L (dNm)	0.4	0.4	0.4	0.3
M_H (dNm)	2.6	4.5	5.1	5.3
ΔM ($\Delta M = M_H - M_L$)	2.2	4.1	4.7	5.0
CR (dNm/min)	0.08	0.2	0.5	0.06
t_{90} (min)	48	31	25	85

Table S2. Tensile properties of covalent and ionic compounds.

	Compounds			
**Parameters**	**2.5ZnO**	**5ZnO**	**10ZnO**	**1S**
M_{100} (MPa)	1.0 ± 0.1	1.9 ± 0.1	2.10 ± 0.04	0.69 ± 0.03
M_{300} (MPa)	1.8 ± 0.1	4.9 ± 0.4	7.6 ± 0.1	0.87 ± 0.02
σ_b (MPa)	9 ± 2	27 ± 7	28 ± 5	4.4 ± 0.7
ε_b (%)	600 ± 61	538 ± 59	472 ± 33	829 ± 54
$\nu \cdot 10^{-5}$ (mol/cm^3)	2.50 ± 0.02	3.1 ± 0.2	3.69 ± 0.03	3.87 ± 0.02

Table S3. Rheometric properties for dual network compounds.

	Compounds		
**Parameters**	**1S-2.5ZnO**	**1S-5ZnO**	**1S-10ZnO**
M_L (dNm)	0.3	0.4	0.4
M_H (dNm)	7.1	8.2	8.9
ΔM ($\Delta M = M_H - M_L$)	6.8	7.8	8.4
CR (dNm/min)	0.2	0.2	0.5
t_{90} (min)	42	53	45

Table S4. Tensile properties of dual network compounds.

	Compounds		
**Parameters**	**1S-2.5ZnO**	**1S-5ZnO**	**1S-10ZnO**
M_{100} (MPa)	1.2 ± 0.1	1.91 ± 0.03	2.8 ± 0.1
M_{300} (MPa)	3.0 ± 0.1	5.0 ± 0.2	11.6 ± 0.4
σ_b (MPa)	8 ± 1	10 ± 1	21 ± 2
ε_b (%)	490 ± 30	394 ± 19	370 ± 11
$\nu \cdot 10^{-5}$ (mol/cm^3)	6.6 ± 0.1	8.2 ± 0.1	9.0 ± 0.1

Table S5. HN fitting parameters of compounds at selected temperature.

	Compounds		
**Parameters**	**1S**	**10ZnO**	**1S-10ZnO**
β – relaxation at -30 °C			
σ_0 (S/cm)	$8.26 \cdot 10^{-16}$	$4.83 \cdot 10^{-16}$	$2.32 \cdot 10^{-15}$
$\Delta\varepsilon$	0.87	0.79	0.84
τ_{max} (s)	$6.54 \cdot 10^{-6}$	$4.04 \cdot 10^{-6}$	$4.45 \cdot 10^{-6}$
τ_{HN} (s)	$2.50 \cdot 10^{-5}$	$1.85 \cdot 10^{-5}$	$1.77 \cdot 10^{-5}$
α	0.38	0.41	0.39
β	0.60	0.52	0.57
α – relaxation at 0 °C			
σ_0 (S/cm)	$3.62 \cdot 10^{-13}$	$2.30 \cdot 10^{-12}$	$1.59 \cdot 10^{-12}$
$\Delta\varepsilon$	10.16	8.52	10.57
τ_{max} (s)	$1.35 \cdot 10^{-4}$	$8.70 \cdot 10^{-5}$	$4.40 \cdot 10^{-4}$
τ_{HN} (s)	$4.12 \cdot 10^{-4}$	$3.54 \cdot 10^{-4}$	$1.95 \cdot 10^{-3}$
α	0.53	0.44	0.42
β	0.53	0.52	0.52
α' – relaxation at 35 °C			
σ_0 (S/cm)	-	$2.62 \cdot 10^{-10}$	$3.30 \cdot 10^{-10}$
$\Delta\varepsilon_{HN1}$	-	21.36	25.25
$\tau_{max\ HN1}$ (s)	-	$7.79 \cdot 10^{-3}$	0.0017
τ_{HN1} (s)		0.001	0.0025
α_{HN1}	-	1	1
β_{HN1}	-	0.72	0.6
$\Delta\varepsilon_{HN2}$	-	10.12	10.58
$\tau_{max\ HN2}$ (s)	-	$3.05 \cdot 10^{-8}$	$9.46 \cdot 10^{-8}$
τ_{HN2} (s)	-	$3.05 \cdot 10^{-8}$	$1.96 \cdot 10^{-7}$
α_{HN2}	-	0.45	0.48
β_{HN2}	-	1	0.69
